# Large Spontaneous Right Catamenial Pneumothorax with Diaphragmatic Defect and Liver Herniation

**DOI:** 10.1155/2019/8436450

**Published:** 2019-05-27

**Authors:** Venkata Kishore Mukku, Emily Cassidy, Catalina Negulescu, Tonya Jagneaux, John Godke

**Affiliations:** ^1^Department of Internal Medicine, Baton Rouge General Internal Medicine Residency Program, an affiliate of Tulane University School of Medicine, Baton Rouge, Louisiana, USA; ^2^Cardiovascular Thoracic Surgery, CVT Surgical Center, Baton Rouge, Louisiana, USA; ^3^Pulmonary Critical Care, Louisiana State University Health Sciences Center, Baton Rouge Campus, Louisiana, USA

## Abstract

Catamenial pneumothorax is a spontaneous pneumothorax that occurs predominantly women of child bearing age. We describe a case of a 40-year-old nulliparous woman with medical history significant for endometriosis who presented with severe chest tightness of one-day duration. Chest radiography (CXR) showed a large right spontaneous pneumothorax, what was thought to be a 5.6 cm pleural mass at the right lung base. Following pneumothorax diagnosis, the patient underwent emergent right thoracostomy with pigtail catheter placement. A repeat CXR revealed marked re-expansion of the lung but persistence of a right pleural mass. Follow up computed tomography scan of the chest showed a 33 mm diaphragmatic defect with 5.8 x 4.6 x 3.9 cm area of herniated liver corresponding to the presumed pleural mass. Following complete thoracic imaging, patient underwent video-assisted thoracoscopic surgery, mechanical pleurodesis, and open repair of the right diaphragmatic defect. Intraoperatively, an endometrial implant was noted on the chest wall. On postoperative day three, she began her menstrual cycle and was evaluated by gynecologist who recommended hormonal therapy to reduce risk of recurrent pneumothorax. Due to a persistent air leak, the chest tube was transitioned to a Heimlich valve to facilitate home discharge. The patient was discharged on postoperative day eight, seen as outpatient with resolution of air leak and removal of chest tube.

## 1. Introduction

Catamenial pneumothorax is a rare cause of spontaneous pneumothorax that affects predominantly women of child bearing age [1]. In the literature, only a few cases of catamenial pneumothorax with diaphragmatic defect and liver herniation are reported [2–7]. In some of these cases, liver herniation was only diagnosed intraoperatively [2–6]. In a case report by Tomescot et al., a chest X-ray (CXR) showed numerous diaphragmatic defects with liver herniation prior to surgical exploration [7]. However, in all these cases liver herniation occurred after recurrence of the pneumothorax. In our case, liver herniation occurred during the first episode of pneumothorax. A computed tomography scan (CT) confirmed liver herniation of what originally appeared to be a pleural based mass on CXR.

Catamenial pneumothorax is most common in premenopausal women aged 30–50 years, with a peak incidence between 30 and 35 years [8]. Catamenial pneumothorax usually occurs within 72-96 hours of onset of menstruation [9]. Catamenial pneumothorax is associated with pelvic endometriosis in 30-50% of cases [10] and almost always involves the right thorax [11]. The etiology of catamenial pneumothorax is poorly understood but several proposed theories exists: migration of air from peritoneal cavity into the pleural space through pre-existing diaphragmatic defects, retrograde implantation of endometrial tissue through diaphragmatic defects, lymphatic or hematogenous spread of endometrial tissue to visceral pleura, and spontaneous rupture of blebs during hormonal changes [12]. Diaphragmatic defects are correlated with endometriosis and have been seen in 29-66% of catamenial pneumothorax patients [11, 13].

## 2. Case Report

**Figure 1:**
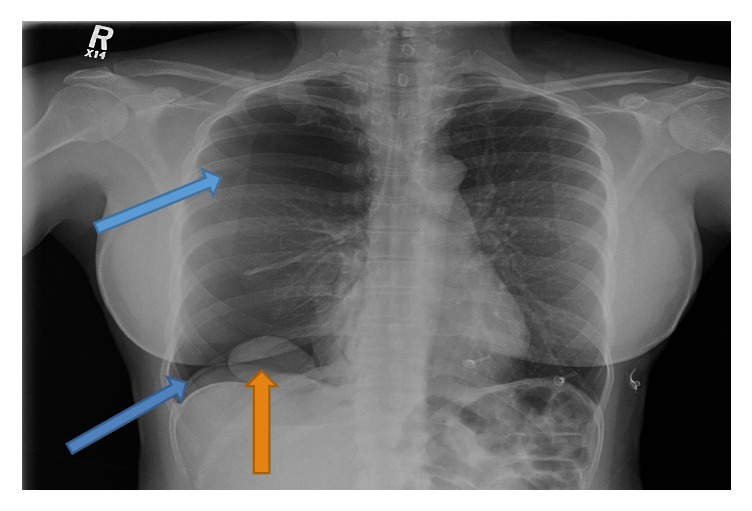
The blue arrows show the visceral pleural line indicating the presence of pneumothorax and diaphragmatic free air. The orange arrow reveals a large 56 mm mass at the base of the lung (confirmed as liver on computed tomography).

A 40-year-old nulliparous woman with no past medical history, other than endometriosis, presented to the emergency room with severe chest tightness of one day duration. She described chest tightness while exercising on a bicycle after 10 minutes. The next day she developed a severe hacking cough at work during a conference call. The cough was associated with disorientation and severe chest tightness. This prompted her to present to the emergency department in February 2018. The patient is a pharmacist, nonsmoker, and denies illicit drug use or recent travel. She had endometrial laser ablation with myomectomy in 2006. Hormonal contraceptives have been used since 2004 and were stopped three months before presentation in hopes of conception. Her last menstrual period was four days before the onset of symptoms. On physical examination, she had chest tightness localizing to the right side and decreased right sided breath sounds. All the routine laboratory work and vital signs were normal. The CXR showed a large right spontaneous pneumothorax with what approved to be a 5.6 cm pleural mass at the right lung base (Figure 1).

**Figure 2:**
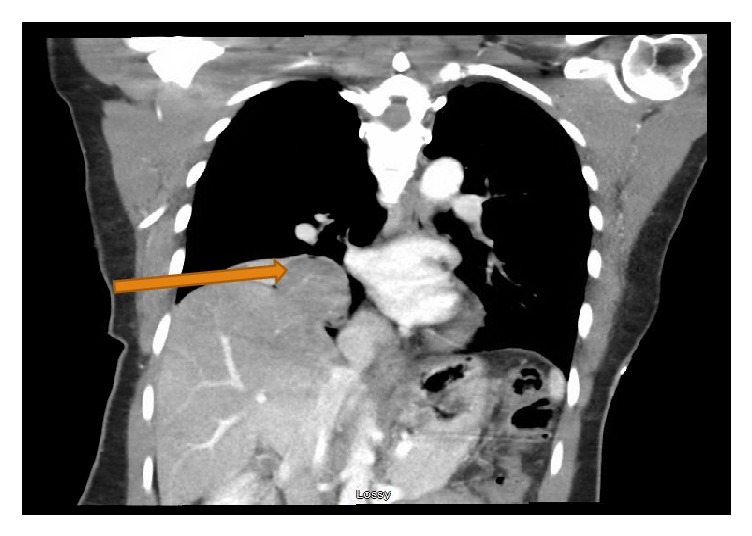
Coronal view of CT scan with orange arrow showing a 33 mm diaphragmatic defect with liver herniation, 5.8 x 4.6 x 3.9cm.

Following the pneumothorax diagnosis, the patient underwent emergent right thoracostomy with pigtail catheter placement. A repeat CXR revealed marked re-expansion of the lung but persistence of the right cardiophrenic opacity of unclear etiology. A follow-up CTof chest showed a 33 mm diaphragmatic defect with a 5.8 x 4.6 x 3.9 cm area of herniated liver corresponding to the presumed pleural mass (Figure 2).

**Figure 3:**
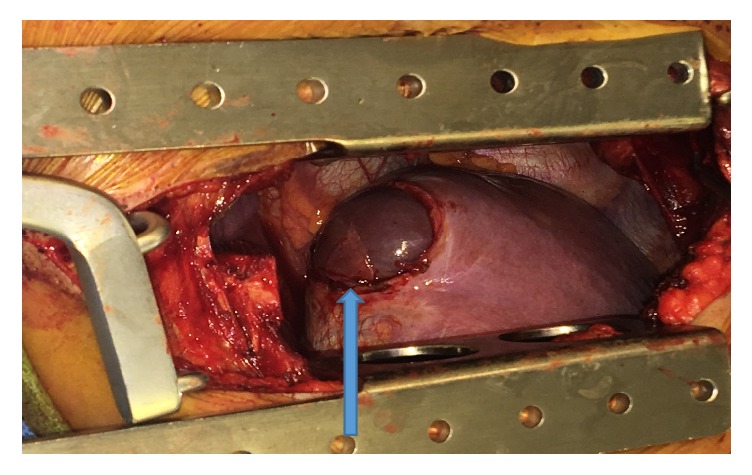
Blue arrow showing large diaphragmatic defect with herniation of liver before repair.

**Figure 4:**
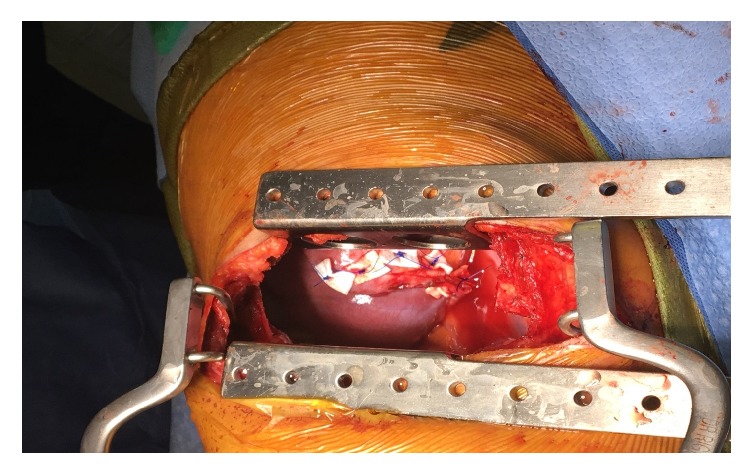
Suture repair of the diaphragmatic defect.

Following complete thoracic imaging the patient underwent video-assisted thoracoscopic surgery (VATS), mechanical pleurodesis, and open repair of the right diaphragmatic defect by Dr. Emily Cassidy (Figures 3 and 4). Intraoperatively, the lungs appeared grossly normal. An obvious diaphragmatic defect was noted in the posteromedial portion of the central tendon of the diaphragm with a sizable protrusion of liver in to the chest cavity. There was an attempt to dissect liver adhesions from the diaphragm. Extensive liver adhesions forced conversion of the right VATS to a posterior lateral muscle sparing thoracotomy. Electrocautery and blunt dissection were used to separate the liver and diaphragm. Once the liver hernia was completely reduced, the diaphragm was repaired using multiple interrupted prolene pledgeted horizontal mattress sutures. Later, right parietal pleurectomy was performed by scoring the pleura posteriorly, anteriorly throughout the entire chest cavity. At the apex as well as the base, where pleurectomy was difficult, mechanical pleurodesis using a Bovie scratch pad was perfomred. A 24-french chest tube was placed at the apex of the chest cavity and 24-french blake was placed at the level of diaphragm.

**Figure 5:**
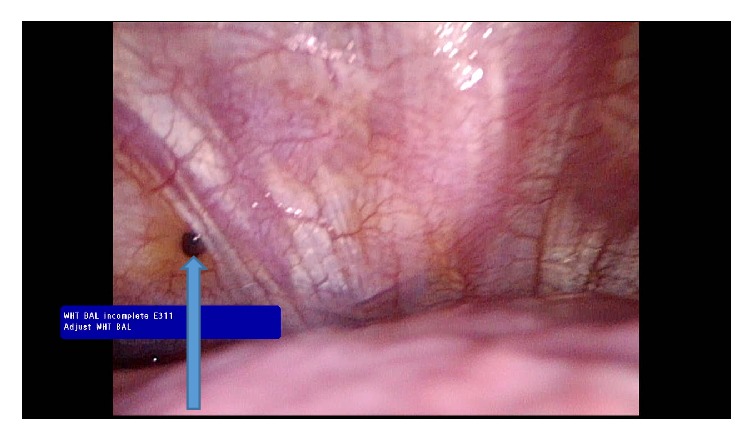
Blue arrow showing an endometrial implant on the chest wall.

Intraoperatively, an endometrial implant (blue berry spot) was noted on the chest wall (Figure 5), but the endometrial implant was not successfully biopsied for pathology evaluation due to the extent of liver adhesions and due to conversion of procedure from VATS to open thoracotomy.

On postoperative day three, the patient began her menstrual cycle. She was evaluated by a gynecologist consultant who recommended hormonal therapy, leuprolide a Gonadotropin releasing hormone (GnRH) analogue, to begin 2-3 weeks postoperatively for a period of 6-12 months for hormonal suppression to reduce the risk of recurrent pneumothorax. GnRH analogs are highly effective at suppressing ovarian hormone production and inhibition of growth of endometrial tissue. Due to a persistent air leak, the patient's chest tube was transitioned to a Heimlich valve to facilitate home discharge. We believed that the persistent air leak indicated there was some minor defect in the visceral pleura that was too small to identify intraoperatively. The patient was discharged on postoperative day eight and was seen as an outpatient by the cardiothoracic surgeon. At this time, there was resolution of air leak and removal of the chest tube. Patient was also seen after two months in a primary care clinic for follow-up visit with no issues.

## 3. Discussion

Though uncommon, this case describes a young woman with endometriosis who presented with pneumothorax and a large diaphragmatic defect. Even though histologic confirmation is lacking, her clinical course, intraoperative finding of endometrial implant, and diaphragmatic defect with liver herniation are consistent with catamenial pneumothorax. She underwent VATS and mechanical pleurodesis with open diaphragmatic repair followed by hormonal therapy. VATS should be timed around beginning of menstrual flow to allow maximum visibility of endometriosis implants. Diaphragm, visceral and parietal pleura needs to be explored for endometriosis implants and all accessible lesions need to be excised [10]. We suspect catamenial pneumothorax in our case could be likely lymphatic or hematogenous spread of endometrial tissue to visceral pleura through pre-existing diaphragmatic defects and spontaneous rupture of blebs during hormonal changes. In our patient, she had an episode of pneumothorax seven days after menstruation and also had menstruation on postoperative day three. So, it is important to block the hormonal support from the ovary to the existing endometrial tissue, to prevent further seeding. Hormonal therapy such as a Gonadotropin-releasing hormone (GnRH) analogue, oral contraceptive pills, progestational drugs, and danazol will help to induce endometrial hypotrophy. Combination of surgery and hormonal treatment reduces recurrence [1, 11, 12].

## 4. Conclusion

Catamenial pneumothorax has a high rate of recurrence, nearly 30 to 40% [13]. Due to high rate of recurrence, these patients require a more aggressive approach of pleurodesis followed by hormonal treatment. Therefore, in an effort to prevent morbidity/mortality, it is important to consider catamenial pneumothorax in any young female presenting with pneumothorax during menses.
